# Neurophysiological Characteristics of Upper Extremity Neuropathy in Three Young Patients with Mucopolysaccharidosis Type I and II in a Five-Year Observation—A Case Series Study

**DOI:** 10.3390/neurolint18020032

**Published:** 2026-02-11

**Authors:** Agnieszka Wiertel-Krawczuk, Zofia Krawczuk, Juliusz Huber

**Affiliations:** 1 Department of Pathophysiology of Locomotor Organs, Poznań University of Medical Sciences, Wiktor Dega Orthopaedic Institute, 28 Czerwca 1956, No 135/147, 61-545 Poznań, Poland; wiertelkrawczuk@ump.edu.pl; 2 Student Scientific Society of Neurophysiologists, Poznań University of Medical Sciences, 28 Czerwca 1956, No 135/147, 61-545 Poznań, Poland; zosiakrawczuk@gmail.com

**Keywords:** mucopolysaccharidosis, Hurler syndrome, Hunter syndrome, clinical neurophysiology, neuropathy, electroneurography, electromyography

## Abstract

**Background/Objectives:** To date, few studies have reported the use of neurophysiological testing to assess the long-term progression of functional changes in median and ulnar nerve conduction in children and adolescents with mucopolysaccharidosis (MPS). This case series study aimed to perform an electroneurographic (ENG) assessment of the median and ulnar nerves in three young patients with MPS treated with enzyme replacement therapy (ERT) and hematopoietic stem cell transplantation (HSCT) over a five-year observation period. **Methods:** Bilateral electroneurography of the motor and sensory fibers in the median and ulnar nerves, recording compound muscle action potential (CMAP) and sensory nerve action potential (SNAP), was performed twice in 5-, 7-, and 19-year-old males at two time points: before and five years after the application of ERT and HSCT. **Results:** In three MPS patients with Hurler or Hunter syndrome, ENG studies similarly demonstrated decreased amplitudes and prolonged distal latencies in their CMAP recordings, confirming the bilateral progression of axonal degeneration and demyelinating changes in the distal parts of the median nerves. The SNAP recordings revealed more severe degenerative processes of similar types in the sensory fibers of the median nerves. Nerve conduction studies in the ulnar nerve fibers bilaterally revealed analogous pathologies, but with a lesser degree of progression. **Conclusions:** This study confirms the progression of axonal degeneration and demyelinating changes in the distal parts of the median nerves, which were associated with decreased amplitudes and prolonged distal latencies in the CMAP recordings of the MPS patients. More expressed degeneration processes of a similar type were found in the sensory fibers of the median nerves. Ulnar nerve pathologies of neural conduction are less advanced in patients with Hurler and Hunter syndromes. It seems advisable to implement neurophysiological diagnostics as soon as possible to specify surgical or conservative therapy, which is crucial for maintaining hand function in the case of progressive peripheral neuropathies in patients with MPS. The timing of the treatment and the patient’s age may be factors influencing the effectiveness of treatment.

## 1. Introduction

Mucopolysaccharidoses (MPSs) are a rare group of congenital genetic diseases where the deficiency of specific lysosomal enzymes leads to impaired glycosaminoglycan (GAG) catabolism [1]. The GAG accumulates in many types of tissue and organs, resulting in the clinical symptoms of MPS being a multisystemic disease [2]. Joint contractures in the absence of inflammation symptoms may indicate mucopolysaccharidosis [3]. In the case of MPS I, a shortage or lack of the Glycosidase α-L-iduronidase (IDUA) enzyme results in many clinical symptoms, which rely on the three clinical phenotypes (Hurler, Hurler–Scheie, and Scheie syndromes). Hurler syndrome is the most severe form, while attenuated forms of MPS I, known as Hurler–Scheie syndrome and Scheie syndrome, are characterized by later onset of symptoms, longer life expectancy, and mild or no central nervous system involvement [4]. The most frequent symptoms accompanying MPS I are facial dysmorphisms, corneal clouding, macroglossia, hearing deficiency, respiratory system dysfunction, and cardiomyopathy. Progressive accumulation of GAG also evokes skeletal deformities, affecting motor activity, restricted joint mobility, and rigidity of paraspinal ligaments. The intensity and range of symptoms, including neurological deficits and cognitive and mental impairments, are associated with MPS I severity. Death usually occurs in the second decade of life, although some patients with less severe forms of the disease have survived into their fifth or sixth decade [5,6,7,8]. Mucopolysaccharidosis of the second type (MPS II) is caused by a lysosomal enzyme iduronate-2-sulfatase (IDS) deficiency. The clinical features of MPS II are similar to those that occur in MPS I [9]. Upper respiratory tract dysfunction with recurrent respiratory infections and cardiologic impairments constitutes the primary cause of death; ocular and gastrointestinal manifestations are also particularly common. The main skeletal symptoms include joint stiffness and vertebral and/or rib abnormalities. In addition, the condition often concerns central nervous system abnormalities and the developmental regression of mental and motor activity [5]. Kwon et al. [6] pointed out that in Hunter syndrome, peripheral neuropathy like CTS is a combination of excessive lysosomal storage in the connective tissue of the flexor retinaculum and a distorted anatomy due to underlying bone dysplasia, also present in the forearm, aggravating median nerve compression. The differences in neurological involvement may be due to the increased heparan sulfate (HS) levels in MPS II, because of HS involvement in neuronal development. The characteristic symptoms in the musculoskeletal system for both MPS I and II have been summarized by Morishita and Petty [10], Palmucci et al. [11], and Viskochil et al. [12] as dysostosis multiplex, short stature, joint contractures, carpal tunnel syndrome (CTS), odontoid hypoplasia, atlanto-axial instability, acetabular dysplasia, coxa valga, genu valgum, and trigger fingers. Progressive deformation of the bone tissue and lysosomal storage in the connective tissue, such as flexor retinaculum, tendons, and synovium, are the main factors for evoked peripheral neuropathy like CTS. MPS disorders are associated with a broad spectrum of neurocognitive effects [13]. Hyperactivity and aggressiveness can be present, especially in patients with severe MPS [14], and because of intellectual disability or a young patient age, detecting the typical symptoms of the peripheral neuropathy is particularly difficult. Progressive hand deformity and muscle atrophy can be the first symptoms [15,16,17]. Clinical neurophysiology with electroneurography (ENG) of sensory (SNAP) and motor fibers (CMAP) in the nerves of the upper extremities can be used to detect early signs of compressive neuropathy, allowing for timely intervention to manage the symptoms. Electromyography (EMG) investigates other neurological complications, such as the muscle atrophy that follows the compression of motor nerve fibers. Treatment decisions like surgical intervention or conventional treatment are guided by clinical neurophysiological examination data [18,19,20,21,22]. According to numerous studies, CTS can develop at a particularly early age. Patients with CTS symptoms have been observed as early as seven months of age. It often occurs bilaterally, usually with an asymmetry of symptoms. Cases of ulnar nerve neuropathy in its distal portion at the level of Guyon’s canal have also been reported in patients with MPS. Early detection of neuropathy, as well as the implementation of appropriate treatments like enzyme replacement therapy, can likely delay the rapid progression of the disease and disabilities, including intellectual deficiencies [15,23,24,25,26,27,28].

In the reviewed literature, we found no reports that have precisely assessed the progression of functional changes in the median and ulnar nerves over time in children and adolescent subjects with MPS [26]. While Hurler and Hunter syndromes share neurological and systemic features, ENG recordings are not explicitly described as a distinguishing factor in their diagnosis as the literature focuses on enzymatic and molecular differences and their associated phenotypic characteristics, such as corneal clouding and the specific GAG buildup. Hence, our study aimed to present a neurophysiological assessment of three MPS patients over a five-year follow-up period. Given the progressive nature of the disease and the particularly crucial early detection of the disease, the case series study aimed to analyze the neurophysiological data over this time in the median and ulnar nerves. This case series study focused on documenting the neurophysiological progression of neuropathy rather than evaluating treatment efficacy for the enzyme replacement therapy (ERT) and hematopoietic stem cell transplantation (HSCT), or disease progression patterns, primarily due to the small size of the research group, which was influenced by the rarity of the MPS disease.

## 2. Materials and Methods

**Figure 1:**
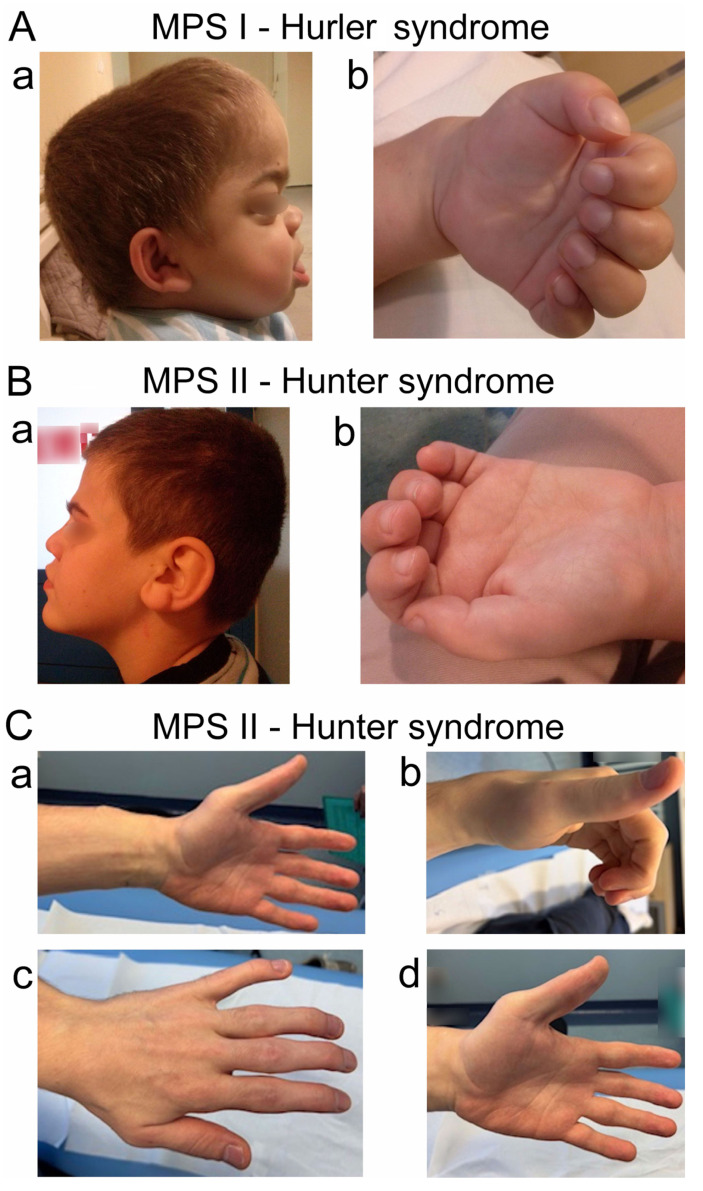
The characteristic phenotypic features of two patients from the study group ((**A**)—MPS I; (**B**)—MPS II). Facial dysmorphisms (**A**(**a**),**B**(**a**)) and a scaphocephalic skull (**A**(**a**)) were especially present in the MPS I patient. Upper extremity deformities like broad (**A**(**b**),**B**(**b**)) and claw hands (**A**(**b**)) were observed. (**C**). Hand deformities (broad hands, joint stiffness, etc.) in a patient with MPS II Hunter syndrome ((**a**,**c**)—first observation; (**b**,**d**)—second observation). Multiple lipomas in the upper extremities and through the anatomical topography of the peripheral nerves were observed (**a**,**d**).

Subjects were examined in the Department of Pathophysiology of Locomotor Organs in the Wiktor Dega Orthopedic and Rehabilitation Hospital of Poznan University of Medical Sciences. Clinical neurophysiology studies were performed on three patients (males) to assess the function of the nerves of the upper limbs in terms of neuropathy. Patients with a previously diagnosed MPS type were referred by the Medical Center that qualified them for enzyme replacement therapy. Clinical neurophysiology studies were one of the inclusion criteria for the therapy. The patients included in the study group were qualified for therapy through clinical assessments at the center. The clinical data presented in Table 1 are derived from the available medical reports. Neurophysiological examinations were performed as part of standard diagnostics once a year for 5 years. All procedures followed the ethical standards detailed within the Helsinki Declaration. Informed consent was obtained from patients or their legal guardian (parent) to be included in this study. Table 1 summarizes the demographic and anthropometric characteristics of the study group and the clinical examination results, with particular emphasis on the neurological symptoms and changes in the skeletal system that may have directly impacted the results of the clinical neurophysiology studies. Figure 1 presents the characteristic phenotypic features of the patients from the study group.

The clinical neurophysiology tests included bilaterally examining the functions of the motor and sensory fibers of the median and ulnar nerves in each patient. The tests were always performed using the same diagnostic equipment by the same experienced neurophysiologists, who had twenty years of experience. The multichannel KeyPoint Diagnostic System (Medtronic A/S, Skøvlunde, Denmark) was used for the CMAP and SNAP recordings in the electroneurography studies (ENG) during the stimulation studies with electrical pulses. For the compound muscle action potential (CMAP) and sensory nerve action potential (SNAP) recordings, a bipolar stimulation electrode and a single rectangular electric stimulus were used at a 1 Hz frequency for 0.2 ms. In the ENG studies, the time base was set to 5 ms/D; the sensitivity of the recordings was 2 mV/D; the upper and lower filters were 10 Hz and 10 kHz, respectively; and a bipolar stimulation electrode was used. The impedance did not exceed 5 kΩ. Skin surface temperature was monitored using a thermometer compatible with the diagnostic device, and it ranged from 30 to 33 °C. Skin temperature range was consistent across all examination periods. If the skin temperature decreased below 30 degrees Celsius, the red light Sollux lamp (PEM, Warsaw, Poland) was used to increase skin temperature, and a second temperature measurement was taken. The air conditioning in the examination room was maintained at 23 degrees Celsius and controlled throughout all examination periods. In the ENG study, the parameters of amplitude (mV) and latency (ms) of the CMAP and SNAP were assessed, and the conduction velocity in these nerves (m/s) was calculated. Table 2 presents a detailed summary of the study group’s clinical neurophysiology methodology.

Data from the ENG parameters of the control group of healthy children used in our laboratory, as described by Garcia et al. [29], were compared with similar recordings obtained from two patients (aged 5 and 7 years old). In the case of the adolescent patient (19 years old), we compared the results with the standard data used in our Department during ENG examinations in adults. In the patients aged 5 and 7 years, the distance between the active electrode on the muscle belly and the first stimulation point proximal to the wrist crease was 5 cm. To evaluate the CMAP parameters and conduction velocity in the median and ulnar nerves of the 3rd patient (19 years old), we used the normative values according to Preston et al. [30] and Holmes et al. [31]. The CMAP distal latency for the median nerve was ≤4.4 ms at an 7 cm distance between the stimulation point at the wrist and the active electrode on the muscle; for the ulnar nerve, it was ≤3.3 ms. Amplitude values for the median nerve were ≥4.0 mV, and for the ulnar nerve were ≥6.0 mV, while the segmental conduction velocity at the forearm and upper arm was ≥50 m/s for both nerves. We aimed to identify statistically significant abnormalities in the nerve impulse conduction in median and ulnar nerve fibers (Table 3).

In the case of the axonal type of motor nerve fiber injury, a needle electromyography was recorded to estimate the current status of the muscle’s bioelectrical activity. We analyzed the spontaneous activity at rest (recordings of fibrillations and positive sharp waves as a factor of active denervation process), the parameters of motor unit action potentials (MUAPs), and the frequency of MUAP recruitment during a muscle’s maximal voluntary contraction [32].

Further methodological details of ENG research are described elsewhere [33,34,35,36].

## 3. Results

Table 3 presents the latency and amplitude values of CMAP and SNAP from the median and ulnar nerves recorded during the first examination in three patients from the research group (one MPS I and two MPS II). The data in the table refer to the symptomatic side, where functional changes in the nerves recorded during the ENG study were more pronounced than on the contralateral side and compared with normative values.

Due to the nature of the report and the small number of analyzed patients, a detailed statistical analysis of the studied parameters is not possible; therefore, we decided to present the data descriptively in analytical longitudinal form without quantitative temporal trend analysis.

**Figure 2:**
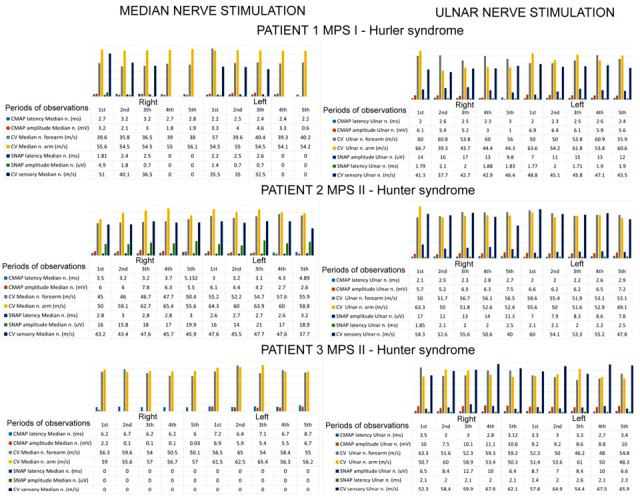
Summary of the results of the bilateral analysis of the CMAP and SNAP parameters from the median and ulnar nerves in the three patients of the research group over a five-year observation period.

**Figure 3:**
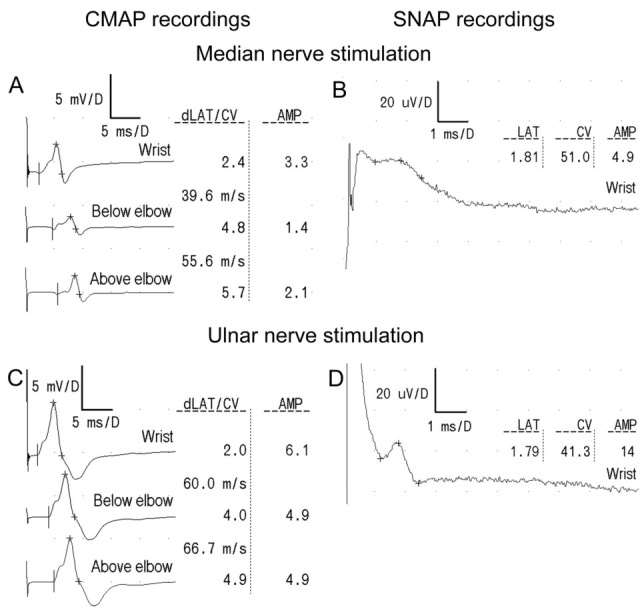
Examples of the CMAP (**A**,**C**) and SNAP (**B**,**D**) recordings following the median (**A**,**B**) and ulnar nerve (**C**,**D**) stimulations in one of MPS II patients. Note the increased CMAP distal latency and slowing down of the conduction velocity in the forearm within the fibers of the median nerve. The time bases in (**A**,**C**), as well as in (**B**,**D**), are similar. The latencies of the recordings are scaled in ms, the conduction velocities are in m/s, and the amplitudes in milli- (**A**,**C**) or microvolts (**B**,**D**). Abbreviations: CMAP—compound muscle action potential; SNAP—sensory nerve action potential; LAT or dLAT—distal latency; CV—conduction velocity; and AMP—amplitude.

Figure 2 consists of a compilation of the ENG data recorded from three patients in the motor and sensory fibers of their median and ulnar nerves, while Figure 3 presents the original ENG recordings from one of the MPS II patients. In Figure 2, parametric values expressed as the descriptive analysis of the clinical neurophysiology tests results in the patients of the research group and analysis of their variability over the 5-year observation period are presented.

During the neurophysiological examination, muscle atrophy was visually assessed and classified as mild (+), moderate (++), and severe (+++). Due to the intellectual disability of the 5- and 7-year-old boys, information regarding daily functioning, impaired muscle strength and motor skills, and possible sensory impairments was obtained from the data provided by their parents. Their intellectual disability also limited muscle assessments that use needle EMG. Due to the lack of required cooperation, this type of study was not performed. In this case, the neurophysiological assessment was incomplete. Subjective evaluation of sensory impairment, fine hand motor skills, and EMG examination were possible in the adolescent patient, as shown in Table 1.

### 3.1. Neurophysiological Findings—Median and Ulnar Nerve CMAP Analysis

In the first patient with Hurler syndrome, a prolonged CMAP distal latency on the right side and a slower bilateral conduction velocity at the forearm in the median nerves were recorded in all five examinations. During the 5-year observation period, a decrease in CMAP amplitude was recorded, indicating a progressive bilateral loss of active motor fibers in the median nerves, confirming the axonal type of nerve injury. In this patient, a progressive decrease in CMAP amplitude was also observed from the right ulnar nerve, with a slower conduction velocity at the elbow level.

In the second patient with Hunter syndrome, a prolonged CMAP distal latency was bilaterally recorded in the median nerves. On the left, the median nerve CMAP amplitude was reduced, indicating an axonal type of nerve damage.

In the third patient with Hunter syndrome, in whom enzyme treatment was started latest (at the age of 12), a significant prolongation of the CMAP end latency from the median nerves was bilaterally recorded, and a progressive reduction in the CMAP amplitude was recorded on the right side.

### 3.2. Neurophysiological Findings—Median and Ulnar Nerve SNAP Analysis

In the first patient with Hurler syndrome functional changes in the sensory fibers showed greater dynamics during the observation periods. A progressively slower conduction velocity and SNAP amplitude reduction were recorded. In the last two years of observation, SNAP was not bilaterally recorded in the median nerves. The slower conduction velocity was bilaterally recorded in the sensory fibers of the ulnar nerves in each observation period.

In the second patient with Hunter syndrome a lower conduction velocity was bilaterally recorded in the sensory fibers of the median nerves. In this patient, a progression of the SNAP latency extension was recorded in the ulnar nerves over a 5-year follow-up period, and there was a slower conduction velocity in the last bilateral examination.

In the third patient with Hunter syndrome, no SNAP was bilaterally recorded from the median nerves during the 5-year follow-up period. Neural transmission in the range of motor and sensory fibers of the ulnar nerves bilaterally remained within the reference values.

### 3.3. Neurophysiological Findings—Needle Electromyography

Due to motor fibers’ axonal loss (detected by reduced CMAP in ENG examination), active denervation and neurogenic changes in muscles can occur. The intellectual disability of the first and second patients, as well as the lack of cooperation and taking anti-coagulants, did not allow for the performance of needle electromyography (nEMG) from the abductor pollicis brevis muscle and the first interosseous muscle. In these patients, we observed the mild atrophy of these muscles.

In the third patient with Hunter syndrome due to the loss of active axons in the motor fibers of the median nerve on the right side, needle electromyography (nEMG) from the abductor pollicis brevis muscle was performed. Chronic neurogenic damage to this muscle was recorded with moderately expressed reinnervation and mild atrophy in this effector.

The neurophysiological test results in cases of axonal damage to the motor fibers of the studied nerves correlated with muscle atrophy. The presence of functional changes in the sensory fibers of the studied nerves in ENG studies did not directly correlate with the patients’ subjective assessment of sensory disturbances, and this was due to the significantly limited ability to interact with patients as a result of intellectual disability. In the adolescent patient, neurophysiological test results correlated with reported sensory loss (consistent with the topography of the innervation pattern) and with weakened muscle strength in the thenar muscles.

## 4. Discussion

### 4.1. Neurophysiological Findings

The most important findings of the current case series study are the confirmation of progressive axonal degeneration and demyelinating changes in the distal parts of the median nerves, which were associated with decreased amplitudes and prolonged distal latency parameters in the CMAP recordings. Analysis of the SNAP recordings provided evidence of the more expressed degeneration processes in the sensory fibers of the median nerves. Similar pathologies were expressed less in the ulnar nerves, which were not described in the studies of other authors. These pathological processes progressed in the long-term observations despite the applied ERT and HSCT therapies.

GAG accumulates in many types of tissue and organs, and it causes the clinical character of MPS multisystemic damage. Deficiency of the proper enzymes contributes, inter alia, to musculoskeletal defects in MPS patients. Similarly, like in our study, joint stiffness, contractures, and significant joint pain are early and prominent signs and are among the earliest manifestations of MPS [10,12]. Previous long-term observation studies, with their generalized influence on multiorgan pathologies in these cases [37], have provided evidence on the efficacy of treatments for MPS when using enzyme replacement therapy and hematopoietic stem cell transplantation. The assessment methods of these prior studies were biochemical and involved neurological tests, but no comparative neurophysiological recordings were presented, which is the novel contribution of the present work.

### 4.2. Clinical Implications

We focused on the neurophysiological assessment of possible neuropathies in MPS that do not result directly from GAG accumulation in the peripheral nervous system, but rather from tissues surrounding the anatomical nerve pathway. Due to skeletal deformity concomitant with MPS, concerning the upper extremities at the wrist and forearm levels, it can provoke peripheral neuropathy in the compression mechanism [38]. Peripheral neuropathy, including carpal tunnel syndrome (CTS) and ulnar nerve neuropathy in the cubital tunnel, is the most frequent type of nerve damage in MPS. The clinical symptoms of the neuropathy in children with MPS may be challenging to identify, can be mild and nonspecific, and it can also be difficult to confirm because of cognitive impairment. In general, the first symptoms of the neuropathy can be recognized by parents and can include manual clumsiness, abnormal manual activity, or a change in playing habits. In our group of patients, the first symptoms of improper hand activity were also recognized by parents. Early detection of the neuropathy is essential in accordance with the surgical treatment. However, after surgical decompression, the conduction of the nerves can persist abnormally [13,19].

Electroneurographic examination can detect early signs of nerve compression, and electromyography can be used to investigate other neurological complications caused by nerve compression and axonal loss, such as the muscle weakness or atrophy in MPS patients [39,40,41]. Maincent et al. [20] recommended annual electroneurography testing for mucopolysaccharidoses patients, starting as early as 3 years of age, including both motor and sensory nerve fiber bilateral transmission in the median and in the ulnar nerves, both at the wrist and the elbow. They examined 13 patients and concluded that surgical intervention can greatly improve the overall function and quality of life of these patients, and it can also prevent irreversible sequelae of median nerve compression. Similarly, Patel et al. [42] analyzed recommendations for CTS treatment and diagnosis in MPS. They concluded that the proper age for screening recommended for conduction studies was at diagnosis, 4 to 5 years of age, but there was a lack of consensus regarding the frequency of screening. Yee et al. [43] indicated that ERT should be initiated as early as possible in patients, recommending the age of 3 years. Zanetti et al. [44], however, suggest that the age of treatment initiation depends on the type of MPS. The severe phenotype, present in about two-thirds of the diagnosed patients, has an earlier onset before 2 years of age. The mild form presents with a later onset, around 3.5–4 years of age. However, the organs’ pathology progressively involved almost all patients, although in the attenuated cases, progression is described as slower.

In our group, the patients did not receive the surgical treatment of the median and ulnar nerve neuropathy; hence, the neurophysiological study during 5 years of observation showed the changes in the median and ulnar nerves that directly resulted from disease progression. The first neurophysiological study of a patient with Hurler syndrome occurred at the age of five. At the same time, ERT was applied to this patient. In one of the patients with Hunter syndrome, the first neurophysiological study was performed at the age of seven, and ERT was administered a year earlier. In the other patient with Hunter syndrome, the first neurophysiological examination was performed at the age of nineteen, and ERT was applied at the age of twelve.

We did not directly evaluate the effectiveness of enzyme replacement therapy or hematopoietic stem cell transplantation when it is administered as recommended for Hurler and Hunter syndromes [45,46]. We indirectly assessed the progression of the disease’s impact on the function of selected peripheral nerves by analyzing nerve conduction parameters over a five-year follow-up period. In the research of White et al. [18,25], ENG anomalies appeared as early as 3 years and 7 months in the youngest child, whereas the average age for clinical symptoms (which can precede clinical signs of CTS) was 6 years. They reported that the first symptoms of CTS occurred, on average, 44.6 months after diagnosing MPS in four examined patients. Given the much earlier onset of ENG anomalies, CTS diagnosis during adolescence is too late [23,24,26]. Our case series studies confirm this assumption. In one of the patients with Hunter syndrome, ERT was initiated late at the age of 12. The severity of median nerve damage was greater compared to the other patients in the study group. Prolonged distal latency parameters in the median nerves and a progressive reduction in CMAP amplitude on the right side over the five-year follow-up were significant in this patient. Atrophy of the APB muscle and the neurogenic nature of its damage were associated with axonal loss, likely secondary to the recorded demyelinating changes in the median nerve. A significant finding in this patient was the absence of bilateral SNAP recordings in the median nerves at all stages of follow-up, indicating the advancement of functional abnormalities. We did not use formal sensory testing scales. The patient reported subjective sensory loss in the distribution of the median nerve innervation. Neurophysiological examination confirmed the reported sensory loss, indicating the loss of active sensory axons. This case suggests that later treatment initiation might correlate with more advanced neuropathy, though this observation requires validation in larger cohorts. Interestingly, with advanced median nerve damage, the CMAP and SNAP parameters from the ulnar nerves were within the normal range at all stages of follow-up. In the other two patients with MPS from the study group, the first examination and treatment were performed earlier. In the case of one of the patients with Hunter syndrome, we observed bilateral prolongation of a terminal latency in the median nerves and a reduction in the CMAP amplitude in the left median nerve. Needle EMG testing was not possible in this patient due to the lack of required cooperation from the child. During the five-year follow-up period, ENG studies also revealed a progressive slowing of the conduction velocity in the sensory fibers of the median nerves in this patient. Progressive prolongation of SNAP latency was also recorded in the ulnar nerves, which resulted in slower conduction velocity in the last two years of follow-up. Contrary to our study, Davis et al. [27] have noted that ulnar ENG evaluations show no compression in the Guyon’s canal in children. However, the present findings show proper median nerve compression with damage in the distal portion of the nerve, initially on the sensory pathway, then becoming motor–sensory. This anomaly was found bilaterally, but the degree of severity was often asymmetrical, similar to our examination. Considering the entrapment of ulnar nerves in the cubital tunnel mentioned by Karpati et al. [47] in familiar Hunter syndrome cases, we observed the ulnar neuropathy in the case of the second patient with Hunter syndrome, where the progressively slower conduction velocity in SNAP and CMAP in all examination periods and decreased CMAP amplitude were recorded. A similar dynamic was bilaterally recorded in the sensory and motor fibers in the median nerves in all examination periods in this patient.

Our case series study showed progressive median and ulnar neuropathy in patients with MPS in a five-year observation period, confirming the developing nature of this disease. Two conditions can predict the role of neuropathy advancement. Mucopolysaccharide deposits in the cytoplasm of fibroblasts may inhibit the formation of regular collagen fibers. Accumulation of mucopolysaccharides within the collagen of tendons, ligaments, and joint capsules may cause nerve compression because of increased tissue tightness along the anatomical course of the nerve, especially in its distal parts. Progressive deformation of the bony structures in the forearms and wrist also contributes to nerve compression and the development of peripheral neuropathies [13]. Our research group did not have any imaging studies confirming skeletal deformities. It is crucial that, in subsequent studies of patients with MPS, the research group be expanded, and conduction parameters be correlated with imaging findings, particularly those concerning the anatomical relationships between the median and ulnar nerves and the surrounding tissues at the distal level of the upper extremity, especially at the anatomical isthmus.

### 4.3. Study Limitations

The main limitation of our study is the small sample size, which is due to the rarity of the disease and the inability of MPS patients to participate in neurophysiological follow-up studies during long-term clinical follow-up. This is also influenced by the high mortality rate of patients with MPS. Because of the small number of analyzed patients, a detailed statistical analysis of the studied parameters was not possible; therefore, we decided to present the data descriptively in analytical longitudinal form without quantitative temporal trend analysis. Another limitation of this study was the inability to validate the standardized functional assessment and scales used in the clinical study due to the lack of this data in the available medical reports of patients from the study group. With the current study group size, patterns of neuropathy progression cannot be determined, which is an area for further expansion of the study group in prospective studies of patients with MPS. The third limitation is the potential for measurement variability, even when the same examiner makes the studies. However, the permissible variation in conduction parameters falls within the normative range, considering the standard deviation. Missing data on treatment compliance and correlation imaging studies with the ENG finding is also curtailment.

## 5. Conclusions

This case series study confirms the progression of axonal degeneration and demyelinating changes in the distal parts of the median nerves, which are associated with decreased amplitudes and prolonged distal latencies in the CMAP recordings of MPS patients. More expressed degeneration processes of a similar type have been found in the sensory fibers of median nerves. Ulnar nerve pathologies of neural conduction are less advanced in patients with Hurley and Hunter syndromes. This study did not directly assess the efficacy of pharmacological treatment or disease progression patterns, primarily due to the small size of the research group, which was influenced by the rarity of the MPS disease. This is the main limitation of this research. Based on our research and that of other authors, it seems advisable to implement neurophysiological diagnostics and appropriate therapy, such as surgical or conservative management, as early as possible, depending on the severity of the disease and the timing of its first symptoms, to maintain hand function in cases of progressive peripheral neuropathies. In the current study, the group size does not allow for determining patterns of neuropathy progression, and assumed hypotheses about disease progression and peripheral nerve affection require validation. This is an area for further expansion of the study group in the prospective research of patients with MPS.

## Figures and Tables

**Table 1:** Summary of the anthropometric data and clinical characteristics of the patients (*n* = 3) in the study group.

Variable	Patient 1	Patient 2	Patient 3
Gender	Male	Male	Male
Age at first ENG examination period (years)	5	7	19
Height at first/last ENG examination period (cm)	110/120	133/158	166/171
Variant of MPS	MPS I Hurler syndrome	MPS II Hunter syndrome	MPS II Hunter syndrome
Enzyme deficiency	alpha-L-iduronidase (IDUA)	iduronate-2-sulfatase (IDS)	iduronate-2- sulfatase (IDS)
ERT/age of first dose (year)	+/5	+/6	+/12
HSCT	+	+	+
Clinical features			
Short posture	1	1	1
Facial dysmorphism and scaphocephalic skull	1	1	1
Skeletal deformities	1	1	1
Upper extremities deformities: claw hands, broad hands	1	1	1
Surgical treatment of CTS	0	0	0
Neurological symptoms:			
Upper extremities sensory abnormalities	1	1	1
Atrophy of hand intrinsic muscles	1	1	1
Wasting and weakness in the hand muscles	1	1	1
Pain syndrome	ND	ND	According to the distribution of the median nerve and lumbo-sacral spinal level
Other features able to cause possible neuropathy	0	0	Lipomatosis
Intellectual disability	1	1	0
Behavior disability—aggressive and auto-aggressive symptoms, psychomotor hyperactivity	0	1	0
Low mood and depressive states	0	0	1

Abbreviations: ENG—electroneurography; ERT—enzyme replacement therapy, HSCT—hematopoietic stem cell transplantation; 1—feature or therapy present; 0—feature or therapy absent; and ND—no data due to a lack of full contact and ability to report pain syndromes by the child.

**Table 2:** Summary of the neurophysiological study methodology.

Median Nerve			Ulnar Nerve	
	CMAP	SNAP	CMAP	SNAP
Recording electrode placement	Abductor pollicis brevis (active electrode on the muscle belly, reference electrode on the muscle tendon)	Middle area of the wrist	Abductor digiti minimi (active electrode on the muscle belly, reference electrode on the muscle tendon)	Medial area of the wrist
Stimulation points	Wrist Cubital fossa Arm	Second and third digit	Wrist Elbow—cubital tunnel Arm	Fifth digit
Electrical stimulus intensity	Individually adapted to achieve supramaximal potentials (from 20 to 100 mA)	20 mA	Individually adapted to achieve supramaximal potentials (from 20 to 100 mA)	20 mA

Abbreviations: CMAP—compound muscle action potential recording; SNAP—sensory nerve action potential.

**Table 3:** A comparison of the results from the median and ulnar nerve electroneurographic studies in groups of healthy young subjects aged 4–6 years (data from Garcia et al. [29], see also Holmes G.L., Moshe S., and Jones R., Clinical Neurophysiology of Infancy, Childhood, and Adolescence, Holmes, G.L. et al., 2005 [31] for elder children and adolescent, and the patients from the study group (n = 3; one MPS I and two MPS II) at different ages. Mean values with standard deviations are presented in healthy groups. The patients’ values of the evoked potential parameters during the first electroneurography study are shown.

Test	ENG Parameter	Controls (Healthy Children Aged 4–6 Years)	Patient 1 (Aged 5 Years) MPS I	Patient 2 (Aged 7 Years) MPS II	Patient 3 (Aged 19 Years) MPS II
		Non-Injured Upper Extremities CMAP *n* = 18 SNAP *n* = 18	Symptomatic Side	Symptomatic Side	Symptomatic Side
Median nerve CMAP	Amplitude (mV)	6.96 ± 2.33	3.2	6.0	2.2
	Latency (ms)	2.56 ± 0.29	2.7	3.5	6.2
Median nerve SNAP	Amplitude (µV)	14.04 ± 5.99	4.9	16	NP
	SCV (m/s)	50.72 ± 3.6	51.0	43.2	NP
Ulnar nerve CMAP	Amplitude (mV)	5.50 ± 1.55	6.1	5.7	10.0
	Latency (ms)	2.03 ± 0.25	2.0	2.1	3.5
Ulnar nerve SNAP (SCV)	Amplitude (µV)	7.35 ± 2.12	14.0	17.0	6.5
	SCV (m/s)	50.25 ± 3.0	37.7	54.3	52.3

Abbreviations: CMAP—compound muscle action potential recording; SNAP—sensory nerve action potential; SCV—sensory conduction velocity; NP—no potential recorded; and ENG—electroneuronography.

## Data Availability

The original contributions presented in this study are included in the article. Further inquiries can be directed to the corresponding author.
